# Genome Sequence of *Pseudomonas putida* Strain ASAD, an Acetylsalicylic Acid-Degrading Bacterium

**DOI:** 10.1128/genomeA.01169-17

**Published:** 2017-10-12

**Authors:** Rebecca E. Parales, Rachita Navara, Rebecca Gettys, Jean J. Huang

**Affiliations:** a Department of Microbiology and Molecular Genetics, University of California, Davis, California, USA; b Franklin W. Olin College of Engineering, Needham, Massachusetts, USA

## Abstract

*Pseudomonas putida* strain ASAD was isolated from compost because of its ability to utilize aspirin (acetylsalicylic acid) as a carbon and energy source. We report the draft genome sequence of strain ASAD, with an estimated length of 6.9 Mb. Study of this isolate will provide insight into the aspirin biodegradation pathway.

## GENOME ANNOUNCEMENT

Pharmaceutical chemicals are frequently detected in municipal sewage, and only some are efficiently degraded during wastewater treatment (1, 2). In one study, of 18 pharmaceuticals measured, aspirin (acetylsalicylic acid) was most abundant in the influent, and it was efficiently degraded during standard primary and secondary treatment (2). To date, however, only one pure culture has been reported to degrade aspirin (3, 4). We isolated *Pseudomonas putida* strain ASAD from compost soil using minimal medium (5) containing acetylsalicylic acid as the sole carbon and energy source. *P. putida* ASAD grew on acetylsalicylic acid with a doubling time of 1.8 h, and preliminary analyses demonstrated that cells are chemotactic to acetylsalicylic acid. Study of this strain can provide insight into how this degradation process occurs and enable exploration of the relationship between degradation and chemotaxis in bioremediation processes.

Genomic DNA was extracted from *P. putida* ASAD using the PowerLyzer PowerSoil DNA isolation kit (Mo Bio) and sequenced to 40× coverage by an Illumina MiSeq instrument at Molecular Research DNA (Shallowater, TX). The genome sequence of *P. putida* ASAD consisted of 6,963,567 bp, with an average GC content of 60.19%. Of the 6,346 predicted genes in the genome, 97% encode proteins of which 79% have a predicted function (6).

*Acinetobacter lwoffii* NCIB 10553 was reported to degrade acetylsalicylic acid using an intracellular esterase that generates acetate and salicylic acid (3, 4), but the gene encoding this enzyme has not been identified. The genome of *P. putida* ASAD contains five annotated acetyl esterases (pput 00474, 00825, 01806, 01976, and 06025), which may be involved in aspirin degradation. In contrast, the genome of *P. putida* F1 (a strain that cannot grow on acetylsalicylic acid) does not carry any acetyl esterase genes. Within the *P. putida* ASAD genome, one salicylate hydroxylase gene (EC 1.14.13.1, GenBank locus OMQ39226, pput 01717) is present. Salicylate hydroxylase catalyzes the formation of catechol from salicylic acid; catechol is then cleaved by a dioxygenase (7). In the same gene cluster encoding the salicylate hydroxylase are genes predicted to encode an aromatic acid transporter, a catechol 1,2-dioxygenase (*catA*), an acyl-CoA reductase, and aryl alcohol dehydrogenase. This putative operon is conserved in the genomes of 3 out of 64 *P. putida* strains examined. At an additional locus, the *P. putida* ASAD genome also carries genes that comprise the β-ketoadipate pathway (8), including a second copy of *catA* that is associated with *catB* and *catC*, as well as *pcaD*, *pcaIJ*, and *pcaF*, which together encode the conversion of catechol to TCA cycle intermediates.

Study of *P. putida* ASAD can determine how it senses acetylsalicylic acid, the enzymes involved in degradation of this compound, and whether these processes are regulated. Strain ASAD has 26 predicted methyl-accepting chemotaxis protein (MCP)-encoding genes. Several studies of pollutant-degrading bacteria such as *P. putida* strains F1 and G7 have shown that genes for chemotaxis to pollutant compounds that are degraded by the bacteria are coordinately regulated with genes for degradation (9). Further study of this strain will provide insight into the sensing and degradation capabilities of *P. putida* ASAD.

### Accession number(s).

This whole-genome shotgun project has been deposited in GenBank under the accession no. MNAH00000000. The version described in this paper is the first version, MNAH01000000. The genome has also been deposited and annotated in the Joint Genome Institute Integrated Microbial Genomes portal (https://img.jgi.doe.gov/cgi-bin/m/main.cgi) with genome ID 2576861821.
